# Corrigendum: Investigating genetic diversity within the most abundant and prevalent non-pathogenic leaf-associated bacteria interacting with *Arabidopsis thaliana* in natural habitats

**DOI:** 10.3389/fmicb.2023.1304377

**Published:** 2023-10-11

**Authors:** Daniela Ramírez-Sánchez, Chrystel Gibelin-Viala, Baptiste Mayjonade, Rémi Duflos, Elodie Belmonte, Vincent Pailler, Claudia Bartoli, Sébastien Carrere, Fabienne Vailleau, Fabrice Roux

**Affiliations:** ^1^LIPME, INRAE, CNRS, Université de Toulouse, Castanet-Tolosan, France; ^2^Gentyane, UMR 1095 GDEC, INRAE, Université Clermont Auvergne, Clermont-Ferrand, France; ^3^Institute for Genetics, Environment and Plant Protection (IGEPP), INRAE, Institut Agro AgroCampus Ouest, Université de Rennes 1, Le Rheu, France

**Keywords:** microbiota, commensal bacteria, genomic diversity, plant growth promotion, growth kinetics, seed inoculation, seedling inoculation, genotype-by-genotype interactions

In the published article, there was an error in the Discussion section. The wording to describe the effect of four bacterial species on plant biostimulation and biocontrol against bacterial pathogens was not precise enough and might have led to some confusion.

A correction has been made to Discussion, *Extensive genetic and genomic diversities within leaf-associated bacterial operational taxonomic units*, second paragraph. Two sentences previously stated:

“Genome sequencing confirmed or refined the *gyrB*-based taxonomic affiliation of four OTUs, i.e., *P. fungorum* (OTU2), *Methylobacterium* sp. (OTU13) and the two *Pseudomonas* species *P. moraviensis* (OTU5) and *P. siliginis* (OTU6). All these four bacterial species have been shown to act as biocontrol agents, to affect root development, to promote vegetative growth and ultimately yield, of diverse plants such as *A. thaliana*, potato, strawberry, tomato ad wheat (Hultberg et al., 2010; Ul Hassan and Bano, 2015; Rafikova et al., 2016; Klikno and Kutschera, 2017; Rahman et al., 2018; Grossi et al., 2020). In addition, both *P. moraviensis* and *P. siliginis* have been identified as the main candidate bacterial species controlling most members of the root and leaf bacterial pathobiota, in particular *P. viridiflava* and *X. campestris*, across natural populations of *A. thaliana* located south-west of France (Bartoli et al., 2018).”

The corrected sentences appear below:

“Genome sequencing confirmed or refined the *gyrB*-based taxonomic affiliation of four OTUs, i.e., *P. fungorum* (OTU2), *Methylobacterium* sp. (OTU13) and the two *Pseudomonas* species *P. moraviensis* (OTU5) and *P. siliginis* (OTU6). All these four bacterial species have been shown to act either as biocontrol agents, or to affect root development, or to promote vegetative growth and ultimately yield, of diverse plants such as *A. thaliana*, potato, strawberry, tomato and wheat (Hultberg et al., 2010; Ul Hassan and Bano, 2015; Rafikova et al., 2016; Klikno and Kutschera, 2017; Rahman et al., 2018; Grossi et al., 2020). In addition, both *P. moraviensis* and *P. siliginis* have been identified in a large consortium of other bacterial species as candidate antagonists of the root and leaf bacterial pathobiota, in particular *P. viridiflava* and *X. campestris*, across natural populations of *A. thaliana* located south-west of France (Bartoli et al., 2018).”

The authors apologize for this error and state that this does not change the scientific conclusions of the article in any way. The original article has been updated.
