# Supplementary material for: Investigating genetic diversity within the most abundant and prevalent non-pathogenic leaf-associated bacteria interacting with Arabidopsis thaliana in natural habitats
Source: Front Microbiol. 2022 Sep 23;13:984832. doi: 10.3389/fmicb.2022.984832 (PMC9537739; doi:10.3389/fmicb.2022.984832)
Supplement: Supplementary file 1 [file Data_Sheet_1.zip › Supplementary Information/Supplementary_information_20220822.docx]

**SUPPLEMENTARY INFORMATION**

**Investigating genetic diversity within the most abundant and prevalent non-pathogenic leaf-associated bacteria interacting with *Arabidopsis thaliana* in natural habitats**

Daniela Ramírez-Sánchez ^1,†^, Chrystel Gibelin-Viala^1,†^, Baptiste Mayjonade^1,†^, Rémi Duflos^1^, Elodie Belmonte^2^, Vincent Pailler^2^, Claudia Bartoli^3^, Sébastien Carrere^1^, Fabienne Vailleau^1,*^ and Fabrice Roux^1,*^

^1^ LIPME, INRAE, CNRS, Université de Toulouse, Castanet-Tolosan, France

^2^ Gentyane, UMR 1095 GDEC, INRAE, Université Clermont Auvergne, Clermont-Ferrand, France

^3^ Institute for Genetics, Environment and Plant Protection (IGEPP), INRAE, Institut Agro AgroCampus Ouest, Université de Rennes 1, Le Rheu, France.

^†^ These authors have contributed equally to this work and share first authorship.

^*^ The authors share senior authorship.

**Primers design strategy**

All the strains with an identity ≥98% for the *gyrase B* sequence of an OTU of interest were considered as belonging to this OTU. Based on this, we BLASTed each *gyrB* OTU sequence against the gyrB database composed of 38,929 sequences (Supplementary Data Set 5) and retrieved all the *gyrB* sequences with an identity ≥ 85%. Subsequently, we split these sequences in two groups, *i.e.* one group composed of sequences with an identity ≥98% for the OTU of interest (named as OTU sequences) and another group containing all the sequences with an identity between 85% and 98% (named as non-OTU sequences). Then, all the sequences were aligned using MUSCLE program (Edgar 2004) and for each polymorphism along the alignment, an allele frequency was calculated for both group of sequences. We retained all polymorphisms with a difference of allele frequency greater than 90% between the two groups of sequences, thereby maximizing inter-group differentiation while maintaining limiting intra-group diversity. Finally, a combination of 2 positions that allows to be specific to the OTU sequence of interest was selected. Primers were designed in a way that these two selected positions were located at their 3’ end, in where mismatches are more likely to have a stronger impact on the specificity. The list of the OTU specific primers are given in Supplementary Table 2.

**Supplementary Figure 1.** CBC and informative-driven approaches to isolate representative strains of the 12 most abundant and prevalent leaf OTUs across 163 natural populations of *Arabidopsis thaliana* located south-west of France.


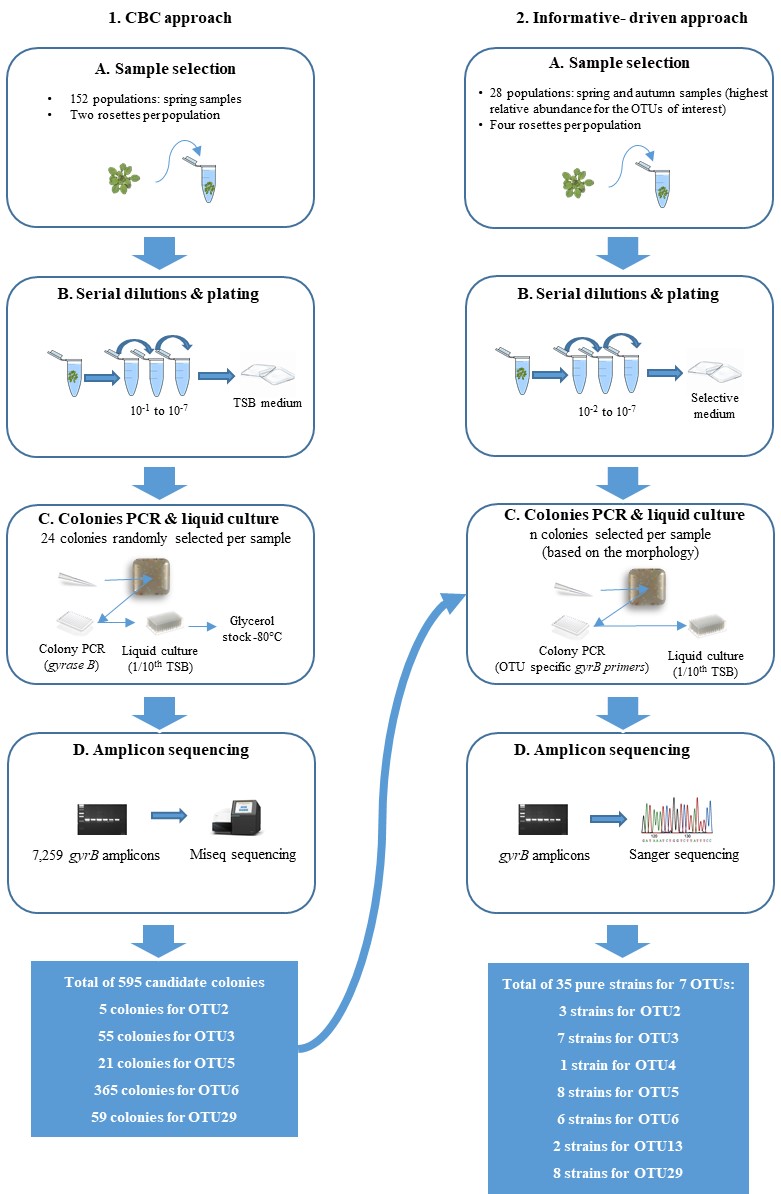


**Supplementary Figure 2.** Pictures illustrating the habitats of the seven accessions chosen to test host genetic variation in response to bacterial isolates.


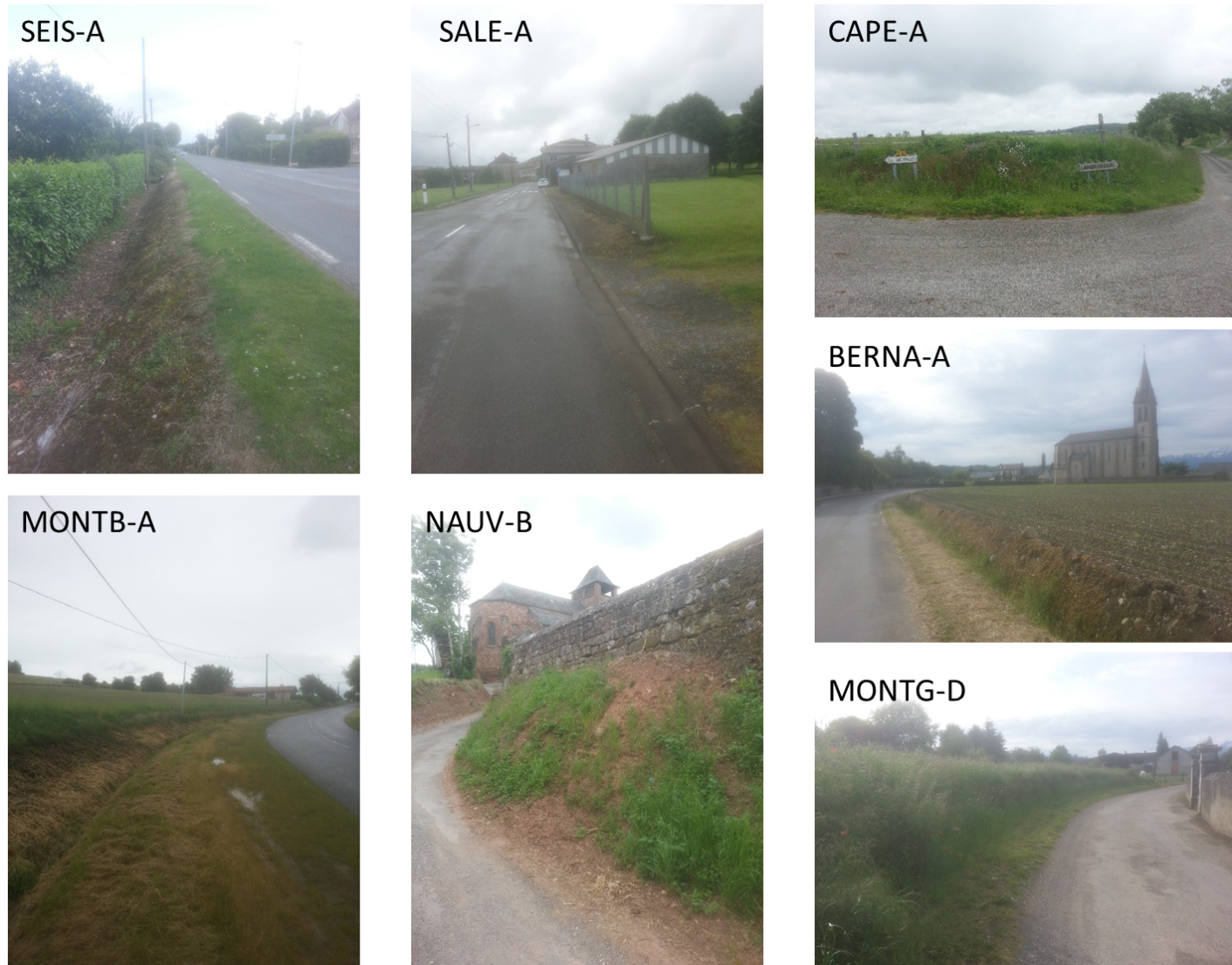


**Supplementary Figure 3.** Intra-OTU genetic variation for growth kinetics on two media with contrasting nutrient availability. **(A)**, **(C)**, **(E)** and **(G)** Intra-OTU genetic variation for the OTUs 4, 5, 13 and 2 on the R2A minimal medium. **(B)**, **(D)**, **(F)** and **(H)** Within-OTU genetic variation for the OTUs 4, 5, 13 and 2 on the TSB rich medium.

**
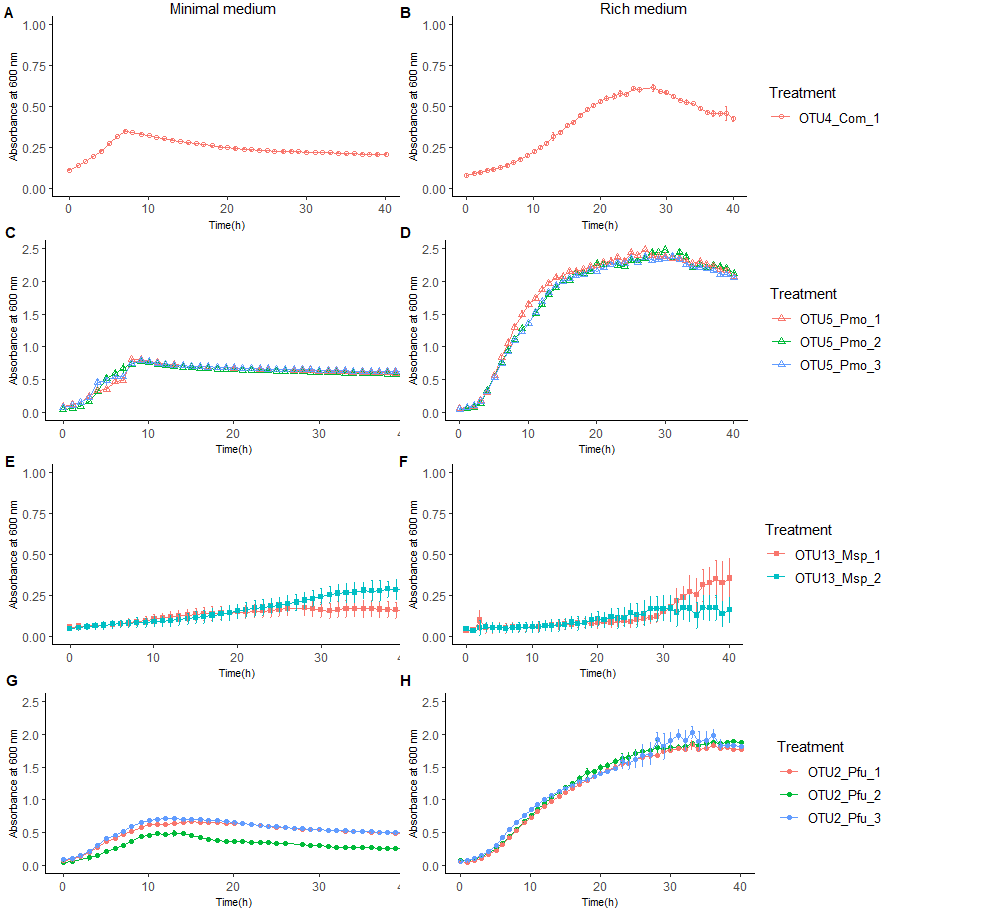
**

**Supplementary Figure 4.** Double hierarchical clustering based on genotypic values, illustrating the genetic variation among the eight *A. thaliana* accessions in response to the 22 representative bacterial strains belonging to eight OTUs at 28 dai. **(A**) Inoculation at the seed stage with scoring at 14 dai (top panel) and 21 dai (bottom panel). **(B**) Inoculation at the seedling stage with scoring at 7 dai (top panel) and 14 dai (bottom panel). Inner plots: Histograms illustrating the distribution of genotypic values for plant development.


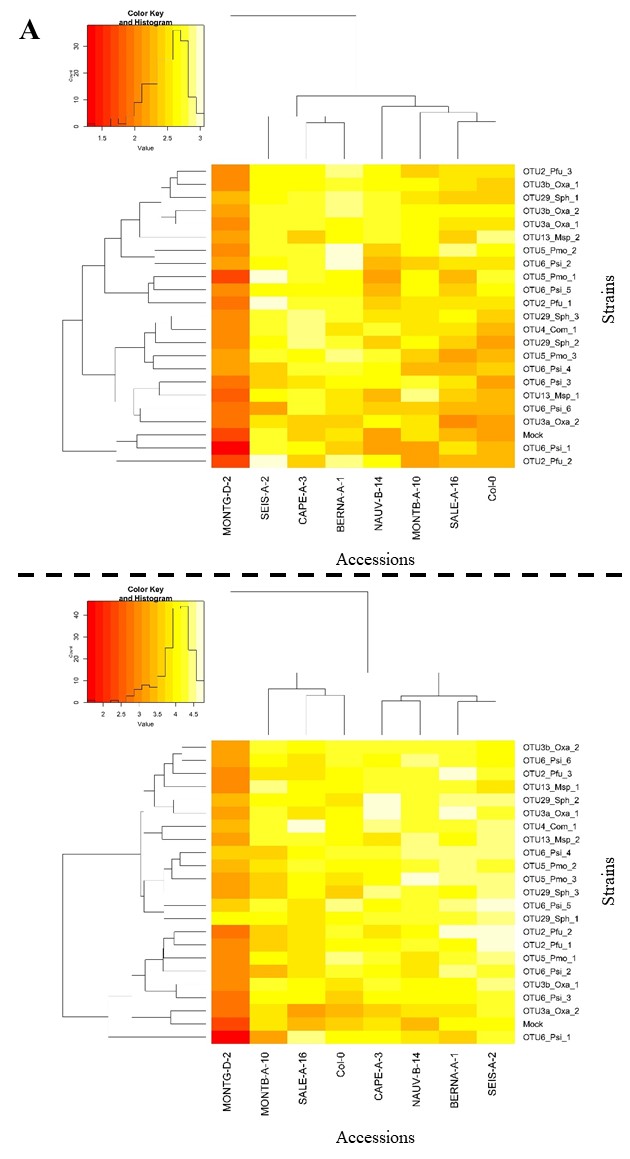


**Supplementary Figure 4 (continued)**


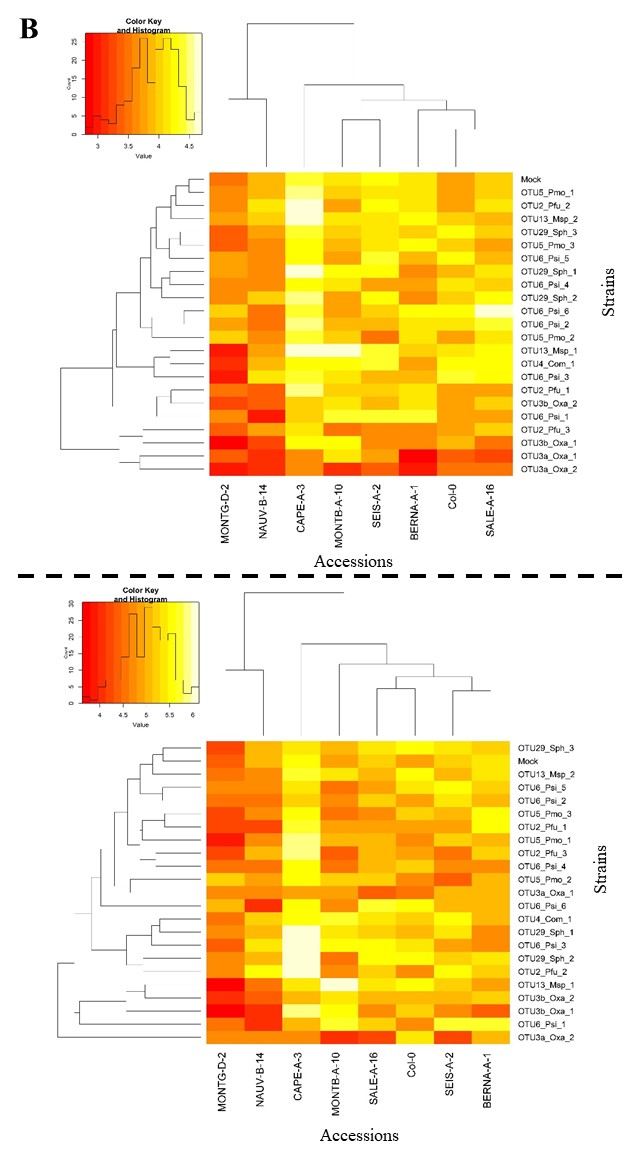


**Supplementary Table 1.** Details about the isolation of the strains characterized in this study. CBC: Community-Based Culture.

* Some strains retrieved from the CBC were not pure and then further purified with selective media.

**Supplementary Table 2.** Primers used to validate the genus level of the OTUs for the informative-driven approach. NA: not available.

**Supplementary Table 3.** *gyrB* primers designed in this study for the informative-driven approach. NA: not available.

**Supplementary Table 4.** ANI values among strains within each OTU. Values highlighted in orange indicate that the strain OTU3b_Oxa_1 belongs to a different bacterial species to which belong the strains OTU3a_Oxa_1 and OTU3a_Oxa_2. Values highlighted in light blue indicate the three strains from OTU5 and the six strains from OTU6 belong to two distinct bacterial species. NE: not estimated

**References**

Bers K, Leroy B, Breugelmans P, Albers P, Lavigne R, Sørensen SR, Aamand J, De Mot R, Wattiez R, Springael D. 2011. A novel hydrolase identified by genomic-proteomic analysis of phenylurea herbicide mineralization by Variovorax sp. strain SRS16. *Appl. Environ. Microbiol.* 77:8754–8764.

Edgar RC. 2004. MUSCLE: multiple sequence alignment with high accuracy and high throughput. *Nucleic Acids Res.* 32:1792–1797.

Fontana PD, Rago AM, Fontana CA, Vignolo GM, Cocconcelli PS, Mariotti JA. 2013. Isolation and genetic characterization of Acidovorax avenae from red stripe infected sugarcane in Northwestern Argentina. *Eur. J. Plant Pathol.* 137:525–534.

Gholami A, Majidpour A, Talebi-Taher M, Boustanshenas M, Adabi M. 2016. PCR-based assay for the rapid and precise distinction of Pseudomonas aeruginosa from other Pseudomonas species recovered from burns patients. *J. Prev. Med. Hyg.* 57:E81-5.

Hwang MSH, Morgan RL, Sarkar SF, Wang PW, Guttman DS. 2005. Phylogenetic characterization of virulence and resistance phenotypes of Pseudomonas syringae. *Appl. Environ. Microbiol.* 71:5182–5191.

Spilker T, Baldwin A, Bumford A, Dowson CG, Mahenthiralingam E, LiPuma JJ. 2009. Expanded multilocus sequence typing for burkholderia species. *J. Clin. Microbiol.* 47:2607–2610.

Yim M-S, Yau YCW, Matlow A, So J-S, Zou J, Flemming CA, Schraft H, Leung KT. 2010. A novel selective growth medium-PCR assay to isolate and detect Sphingomonas in environmental samples. *J. Microbiol. Methods* 82:19–27.
